# Evaluation of the Performance of a Dengue Outbreak Detection Tool for China

**DOI:** 10.1371/journal.pone.0106144

**Published:** 2014-08-29

**Authors:** Honglong Zhang, Zhongjie Li, Shengjie Lai, Archie C. A. Clements, Liping Wang, Wenwu Yin, Hang Zhou, Hongjie Yu, Wenbiao Hu, Weizhong Yang

**Affiliations:** 1 Key Laboratory of Surveillance and Early-warning on infectious Disease, Division of Infectious Diseases, Chinese Center for Disease Control and Prevention, Beijing, China; 2 Research School of Population Health, College of Medicine, Biology and Environment, The Australian National University, Canberra, Australia; 3 Infectious Disease Epidemiology Unit, School of Population Health, University of Queensland, Brisbane, Australia; Institut Pasteur, France

## Abstract

An outbreak detection and response system, using time series moving percentile method based on historical data, in China has been used for identifying dengue fever outbreaks since 2008. For dengue fever outbreaks reported from 2009 to 2012, this system achieved a sensitivity of 100%, a specificity of 99.8% and a median time to detection of 3 days, which indicated that the system was a useful decision tool for dengue fever control and risk-management programs in China.

## Introduction

Detecting infectious disease outbreaks quickly is crucial for timely implementation of control measures, thereby minimizing morbidity and mortality. To automatically identify aberrations in disease incidence data at an early stage, some countries have established infectious disease surveillance and outbreak detection systems, such as the Early Aberration Reporting System (EARS) of the US Centre for Disease Control and Prevention [1], the Real-time Outbreak and Disease Surveillance (RODS) system of the University of Pittsburgh [2], the Electronic Surveillance System for the Early Notification of Community-Based Epidemics (ESSENCE) in the USA [3], and SurvNet@RKI in Germany [4].

Dengue fever (DF) is one of the world’s most important vector-borne diseases, with cases reported from more than 100 countries in Africa, America, Southeast Asia, the Western Pacific and Europe. The World Health Organization estimates that DF affects over 50 million people annually [5]. In 1978, DF re-emerged in Guangdong province, China, after having disappeared from the country for more than 30 years [6]. From 1978 to 2012 more than 650,000 cases of DF, and hundreds of deaths, were documented in China. DF epidemics had spread from Guangdong, Hainan and Guangxi provinces in the southern coastal regions to some relatively northern regions including Fujian and Zhejiang provinces [7]. It has been suggested that establishing an early outbreak detection system is one of the prerequisites for adequate preparedness and responses to DF epidemics [5], which can enable better engagement of the community in prevention and control activities, thereby reducing DF transmission and improving clinical outcomes.

To facilitate early detection of infectious disease outbreaks, the Chinese Centre for Disease Control and Prevention (China CDC) has developed the China Infectious Disease Automated-alert and Response System (CIDARS), implemented since April 2008 [8]. CIDARS automatically conduct the aberration detection from the reported data in the web-based Nationwide Notifiable Infectious Diseases Reporting Information System (NIDRIS). This was established in 2004 and is the largest direct infectious disease reporting system in the world, covering all general hospitals in the prefectures, and all hospitals in the counties and townships in China [9]. CIDARS has been operating in China for more than four years. This study aims to provide a preliminarily prospective evaluation of the performance of CIDARS for DF outbreak detection during the initial phase of real-world implementation nationwide.

## Methods

As a notifiable disease in China, DF are diagnosed by clinicians according to the diagnostic criteria for DF enacted by the Chinese Ministry of Health, which includes suspected cases, clinically diagnosed cases and laboratory-confirmed cases. They are then reported to NIDRIS.

In CIDARS, a time-series moving-percentile method (MPM) was used to detect aberrations of DF occurrence at the county level for the 31 provinces by comparing the reported cases in the current observation period to those of the corresponding historical period for each county. Accounting for the day-of-week effect and the stability of data, we used the most recent seven-day period as the current observation period and the previous three years as the historical period [1], [10]. The number of cases in the current observation period is the sum of reported cases within the recent seven days. The corresponding historical period included, for each of the previous three years, the same seven-day period, the two preceding seven-day periods and the two following seven-day periods, resulting in 15 historical seven-day data blocks covering 105 days. The 50th percentile of the 15 blocks of historical data was set as the threshold value to trigger a signal in CIDARS. The current observation period and historical data blocks were dynamically moved forward day by day [8]. When the number of reported cases in the current observation period reached the defined threshold, CIDARS automatically generated a signal.

According to the operational proposal of CIDARS, action should be taken in response to the signals, which included two steps: (1) signal initial verification, and (2) signal field investigation. The signal initial verification was carried out by epidemiologists in local CDCs by reviewing the reported cases, conducting the rapid assessment of information from other surveillance sources or directly contacting the cases reporting agencies. If the signal denoted one suspected outbreak after the initial verification, then a field investigation was conducted to confirm whether an outbreak was occurring, otherwise this signal would be determined as a negative signal.

The nationwide reported outbreaks from 2009 to 2012 were adopted as the reference standard to evaluate the performance of outbreak detection by CIDARS. According to the national DF surveillance proposal, the definition of a reported DF outbreak was ≥3 cases occurring in a concentrated setting (e.g., community, school or village) within 15 days [11]. All outbreaks defined using this criteria should be reported to an information system in China CDC by the public health staff of local CDC. The start and end dates of an outbreak were defined as the dates when the first and last cases of the outbreak was reported. When CIDARS generated a signal during the period of outbreak occurrence, we considered the outbreak to have been detected.

In this study, we investigated the spatial and temporal patterns of reported DF cases and signals from January 1st, 2009 to December 31st, 2012. Sensitivity, specificity and time to detection (TTD) were employed to assess the performance of CIDARS for early detection of DF outbreaks during the study period [12]. Sensitivity was defined as the number of outbreaks during which at least one signal was triggered by CIDARS, divided by the total number of reported outbreaks. Specificity was defined as the number of non-outbreak days with no signal, divided by the total number of non-outbreak days. TTD was defined as the interval between the start of each outbreak and the first signal during the outbreak. If the signal was triggered on the first day of an outbreak, TTD was zero.

## Results

**Figure 1:**
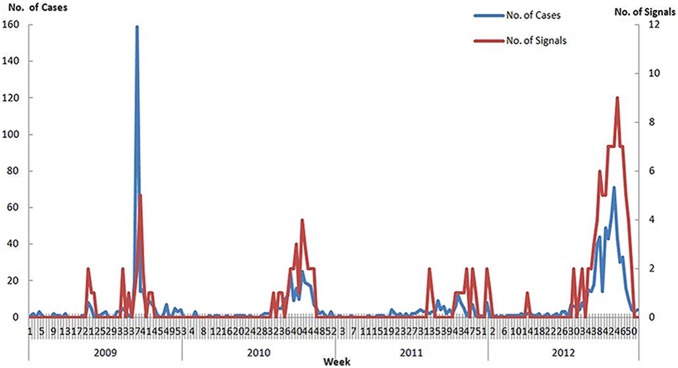
The time distribution of dengue fever cases and signals between 2009 and 2012 in China.

**Figure 2:**
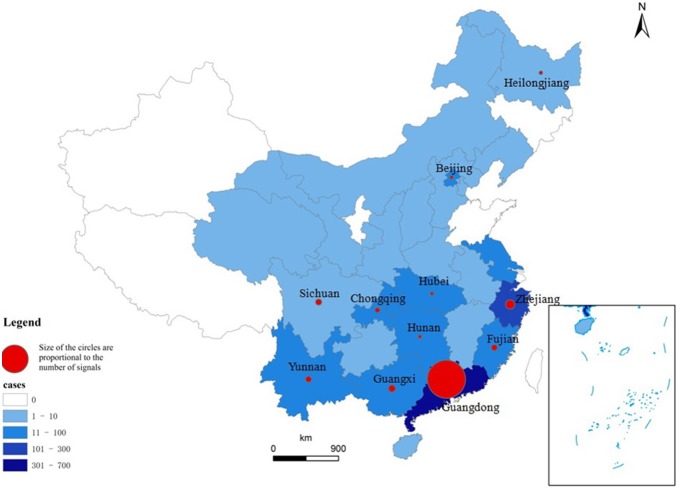
The spatial distribution of dengue fever cases and signals between 2009 and 2012 in China.

Between 2009 and 2012, 1224 DF cases were reported in China. The temporal pattern showed seasonal variation, with 86.8% (1,062) of cases being reported from the 31^st^ week to the 49^th^ week of each year, which corresponds to late summer and autumn in China. The annual average incidence was 0.023 per 100,000 between 2009 and 2012, and the case numbers were higher in 2009 and 2012 (Figure 1). A total of 147 signals for DF outbreaks were generated in CIDARS, of which 91.8% (135) occurred from the 32^nd^ week to the 49^th^ week between 2009 and 2012 (Figure 1), which was consistent with the DF case occurrence. Twenty-six of China’s 31 provinces reported DF cases and signals were generated in 11 provinces (Figure 2). Most cases were reported in Guangdong (682, 55.72%) and Zhejiang (228, 18.63%). Accordingly, most signals were generated in Guangdong (121, 82.31%) and Zhejiang (7, 4.76%), respectively. Overall, there were 30 outbreaks reported from 4 provinces located in the south-eastern coastal regions, including 20 outbreaks in Guangdong, 7 in Zhejiang, 2 in Guangxi and 1 in Fujian (Figure 2), lasting 405 days and including 325 cases. CIDARS successfully detected all the 30 outbreaks, with a sensitivity of 100% and a specificity of 99.8%. The median time to detection (TTD) of all outbreaks was 3 days, with an interquartile range of 4 days (Table 1).

**Table 1:** Dengue fever outbreaks between 2009 and 2012 in China.

OutbreakID	Casesincluded inthe outbreak	Reported dateof the first case(No. of cases on that day)	Reported dateof the last case(No. of cases on that day)	Interval of thefirst and lastreported cases of theoutbreak (days)	Whether or notoutbreak was detected(No. of detection signals)	Date of firstdetection signal	Time to detection(days)
1	3	Aug 8, 2009 (2)	Aug 10, 2009 (1)	3	Y (1)	Aug 10, 2009	3
2	19	Sept 9, 2009 (3)	Sept 21, 2009 (1)	13	Y (2)	Sept 9, 2009	0
3	49	Sept 9, 2009 (2)	Sept 22, 2009 (1)	14	Y (2)	Sept 9, 2009	0
4	100	Sept 9, 2009 (11)	Sept 21, 2009 (3)	13	Y (2)	Sept 9, 2009	0
5	4	Sept 13, 2009 (1)	Sept 17, 2009 (1)	5	Y (1)	Sept 16, 2009	3
6	7	Sept 14, 2009 (7)	Sept 14, 2009 (7)	1	Y (1)	Sept 14, 2009	0
7	3	Sept 19, 2009 (1)	Sept 22, 2009 (2)	4	Y (1)	Sept 23, 2009	4
8	7	Sept 25, 2009 (1)	Oct 9, 2009 (1)	15	Y (1)	Sept 29, 2009	4
9	4	Sept 28, 2009 (1)	Oct 3, 2009 (1)	6	Y (1)	Oct 13, 2009	6
10	4	Sept 30, 2009 (1)	Oct 5, 2009 (1)	6	Y (1)	Oct 3, 2009	3
11	3	Aug 19, 2010 (3)	Aug 19, 2010 (3)	1	Y (1)	Aug 19, 2010	0
12	3	Sept 30, 2009 (1)	Oct 2, 2010 (2)	3	Y (1)	Sept 30, 2009	0
13	9	Oct 14, 2010 (9)	Oct 14, 2010 (9)	1	Y (1)	Oct 14, 2010	0
14	10	Oct 10, 2011 (1)	Oct 21, 2011 (3)	12	Y (2)	Oct 11, 2011	1
15	11	Aug 14, 2012 (1)	Sept 30, 2012 (1)	45	Y (5)	Aug 16, 2012	2
16	4	Sept 11, 2012 (1)	Sept 27, 2012 (1)	17	Y (2)	Sept 17, 2012	6
17	3	Sept 14, 2012 (2)	Sept 15, 2012 (1)	2	Y (1)	Sept 14, 2012	0
18	12	Sept 18, 2012 (1)	Oct 30, 2012 (1)	42	Y (6)	Sept 21, 2012	3
19	9	Sept 20, 2012 (1)	Sept 28, 2012 (1)	9	Y (2)	Sept 21, 2012	1
20	8	Oct 4, 2012 (1)	Nov 9, 2012 (1)	35	Y (6)	Oct 5, 2012	1
21	5	Oct 9, 2012 (1)	Oct 15, 2012 (1)	7	Y (1)	Oct 12, 2012	3
22	5	Oct 9, 2012 (1)	Oct 25, 2012 (1)	17	Y (2)	Oct 15, 2012	6
23	5	Oct 11, 2012 (1)	Oct 25, 2012 (2)	15	Y (2)	Oct 15, 2012	4
24	4	2012-10-13 (1)	2012-10-30 (1)	18	Y (2)	Oct 19, 2012	6
25	8	Oct 13, 2012 (1)	Nov 3, 2012 (1)	22	Y (3)	Oct 19, 2012	6
26	4	Oct 18, 2012 (1)	Nov 16, 2012 (1)	29	Y (4)	Oct 22, 2012	4
27	4	Oct 22, 2012 (1)	Nov 7, 2012 (1)	17	Y (4)	Oct 22, 2012	0
28	3	Oct 23, 2012 (1)	Oct 30, 2012 (2)	8	Y (1)	Oct 26, 2012	3
29	6	Oct 23, 2012 (1)	Oct 30, 2012 (1)	8	Y (1)	Oct 26, 2012	3
30	9	Oct 24, 2012 (1)	Nov 9, 2012 (1)	17	Y (3)	Oct 25, 2012	1

## Discussion

Our study found that CIDARS successfully detected all the DF outbreaks and had a high specificity and timeliness, by adopting a simple and convenient algorithm to automatically generate the early warning signals when aberrations of cases occurred.

In the field of outbreak detection system evaluation, as the true outbreak is nearly impossible to obtain in the real world, the reported outbreaks with high reporting quality are commonly adopted as the reference standards to evaluate the system [4], [13]–[15]. In China, as DF is a notifiable and concerning disease, once getting information on potential outbreaks reported from clinical institutes, media, and community, etc, the public health staff of local CDC immediately launches verification and investigation. Then local CDC reports all the confirmed outbreaks to China CDC according to the outbreak definition issued by Chinese Ministry of Health. Therefore, we supposed the reported outbreaks were the outbreak data source most approximating to the true outbreaks situation, and adopted the reported outbreaks as the reference standard for calculating the sensitivity, specificity and timelines of outbreak detection. Certainly, in practice, it is possible that an outbreak occurred but no cases were reported, which lead to that outbreak would not be captured by CIDARS. Therefore, we regard the quality of case reporting and outbreak reporting as critical to evaluate the performance of CIDARS. By using the reported cases and outbreaks during the 3 years of 2009–2012 in this study, we found that CIDARS could detect all the reported outbreaks during the study period, which were currently regarded as 100% sensitivity of outbreak detection for CIDARS. We admit that, as the number of reported outbreaks during study period is limited, which may not represent the true situation of DF outbreaks in China, more data over a longer time period should be adopted to prospectively validate and evaluate the sensitivity of CIDARS for DF in the future.

As for all early detection tools, the trigger threshold for a signal should be set according to the practical requirements of outbreak detection and response under local conditions. Taking account of the high priority placed on control and prevention of DF in China, with the aim to catch as many outbreaks as possible in the preliminary phase of CIDARS operation, it was decided that the CIDARS needed a relatively low threshold leading to a high sensitivity of the algorithm. Following the advice of senior epidemiologists and the CIDARS research group, the 50th percentile (the median value) of historical data was defined as the trigger threshold, which means that when the current disease incidence level reached the median of corresponding historical baseline incidence level, local public health should be vigilant. Actually, this study was a prospective analysis of a real-world system rather than a retrospective analysis testing different theoretical thresholds. Future evaluation of CIDARS could make use of more cases and outbreaks data collected over a longer period to further assess the performance of CIDARS using different thresholds, helping us optimize the trigger threshold by ROC graph [16] and detection timeliness.

In this study, we found that the timeliness of outbreak detection by CIDARS varied distinctly among outbreaks with similar size, for example, both outbreak ID 9 and 27 have low total number of cases for the entire outbreaks and low number of cases reported on both the first and last days of outbreak, and TTD varied significantly between them. The major cause is that the generation of a signal by CIDARS was affected by many factors, including the current disease incidence level, the spatiotemporal distribution of reported cases, and the incidence level in historical baseline, which may lead to the variation on the timeliness of outbreak detection of CIDARS. We will consider further validating and improving the algorithms of outbreak detection by taking into account various level of dengue incidence.

In this study, all the dengue cases, including imported and local DF cases, were used to detect DF outbreaks. However, for the no local dengue transmission regions where only imported dengue cases occurred, or with few or no indigenous cases, the signals generated by CIDARS may only show the imported dengue occurring, not the dengue fever outbreak in these regions. That may be why the signals were generated in 11 provinces, while DF outbreaks just occurred in 4 provinces which are local dengue transmission regions. One limitation of CIDARS is that it is difficult to predict DF outbreaks before cases are diagnosed and reported, because this system is only based on numbers of notified DF cases. However, it is possible to incorporate vector surveillance data or data pertaining to environmental and social drivers of disease risk in CIDARS using spatiotemporal models, which could lead to a more rapid response than is possible with the current system [17]–[19]. In the future, an early detection system based on socio-environmental factors for DF should be developed and integrated into CIDARS using rigorously evaluated modelling methods and processes.

The findings of this study indicated that CIDARS had good sensitivity, specificity and timeliness of DF outbreak detection, and the system could act as a tool to assist early detection on outbreaks for the local public health staff in China. CIDARS automatically carried out abnormality detection on case occurrence data, and reminded the local CDC to verify aberrations by distributing signals to them. CIDARS assisted the local public health staff to detect potential outbreaks early, and even confirm some outbreaks which passive report did not detect. In future research, we will evaluate the impact of CIDARS on the number and size of dengue outbreaks with more long-term data, so as to show the significance of CIDARS on recognition and response to DF outbreaks.

Mr Honglong Zhang is a Research Intern of Epidemiology and Health Statistics in the Division of Infectious Diseases, Chinese Center for Disease Control and Prevention. He is mainly engaged in the research of disease surveillance and outbreak detection algorithms, and developing the application tools of outbreak early detection.
